# A Case of Prolapsus of the Vagina, Preceding Labor

**Published:** 1885-07

**Authors:** O. O. Simpson

**Affiliations:** Logansville, Ga.


### A CASE OF PROLAPSUS OF THE VAGINA PRECEDING LABOR.

BY O. O. SIMPSON, M. D., LOGANSVILLE, GA.

On the 21st of November last, I was called to see a colored woman, age 35, who had been in labor for fifteen hours. She had been attended during the entire time by a midwife.

On my arrival the midwife informed me that there was "something wrong." She stated that the patient had not urinated for ten hours, and that she thought the afterbirth was trying to come away first.

On examination I found a large tumor presenting at the orifice of the vagina, extending about three inches beyond the labia. This tumor proved to be the prolapsed vagina. The midwife informed me that during the pains the tumor would project five or six inches. After satisfying myself of the nature of the case, I placed the woman in the knee-chest position and replaced the prolapsed vagina. I could then, without difficulty, pass my finger up to the os, which I found dilated very little. The woman was very much exhausted. I placed her in the left lateral position, with her hips slightly elevated, and gave her one-fifth grain of morphia hypodermically. She soon fell asleep and rested for nine hours, when she was aroused by the return of the labor pains. She was kept in the position in which she had been placed until the uterus was well dilated, when she was allowed to turn on her back, and was soon delivered of a fine, healthy child.

This case, to my mind, shows clearly the value of morphia hypodermically in protracted cases of labor.

